# Virtual reality cue exposure as an add-on to smoking cessation group therapy: a randomized controlled trial

**DOI:** 10.1186/s13722-025-00561-2

**Published:** 2025-04-11

**Authors:** A. M. Kroczek, B. Schröder, D. Rosenbaum, A. Mühleck, J. Diemer, A. Mühlberger, A. C. Ehlis, A. Batra

**Affiliations:** 1 Department of Psychiatry and Psychotherapy, Tübingen Center for Mental Health (TüCMH), University Hospital Tübingen, Tübingen, Germany; 2 Department for Psychology, Clinical Psychology and Psychotherapy, University of Regensburg, Regensburg, Germany; 3 Department of Psychiatry and Psychotherapy, Tübingen Center for Mental Health (TüCMH), Section for Addiction Research and Medicine, University Hospital Tübingen, Tübingen, Germany; 4 kbo-Inn-Salzach-Hospital, Wasserburg am Inn, Germany; 5 Department of Psychology, LMU Munich, Munich, Germany; 6 German Center for Mental Health (DZPG), Partner site Tübingen, Tübingen, Germany

**Keywords:** Smokers, Smoking cessation, Cue reactivity, Cue exposure, Craving, Virtual reality

## Abstract

**Background:**

Cue exposure (CE) is used for relapse prevention as part of smoking cessation therapy to reduce the automatized response to smoking-related cues. Using CET in virtual reality (VR) is an approach to increase its efficacy by creating cost-efficient high-risk situations. The efficacy of VR-based CETs was compared to that of an unspecific relaxation intervention as an add-on to an established cognitive-behaviorally oriented smoking cessation group therapy (G-CBT).

**Methods:**

*N* = 246 abstinence-motivated smokers were included in a two-armed randomized controlled trial (G-CBT with VR-CET vs. G-CBT with progressive muscle relaxation/PMR) with 1-, 3-, and 6-month follow-ups (measurements in 2018–2020). All smokers joined a well-established G-CBT smoking cessation program with six sessions with four additional sessions of either VR-based smoking cue exposure therapy (VR-CET) or four sessions of group-based PMR. The primary outcome was abstinence after 6 months according to the Russell Standard; secondary outcomes included changes in the number of smoked cigarettes, craving (assessed by the Questionnaire of Smoking Urges/QSU), and self-efficacy (assessed by the Smoking Abstinence Self-Efficacy Scale/SES) over time.

**Results:**

Primary outcome: Six months after G-CBT, 24% of the participants were abstinent, and there was no significant difference between the PMR (*n* = 34/124) and VR-CET (*n* = 24/122) groups (odds ratio PMR/VR = 0.64). Secondary measures: SES increased, and QSU and the number of smoked cigarettes decreased over time. Baseline craving ratings predicted abstinence only in the VR-CET group.

**Conclusion:**

This randomized controlled trial did not show increased abstinence rates related to smoking cue exposure in virtual reality. Secondary measures demonstrated significant reductions in craving and cigarette consumption as well as increases in self-efficacy over time, regardless of the intervention. However, high baseline craving was negatively related to abstinence in the VR-CET group, suggesting that intense craving was insufficiently addressed. This may indicate that the amount of training was insufficient and should be intensified. Individualization, e.g., adaptive, individualized approaches, is required to improve the effects of the VR-CET on smoking cessation in future studies.

**Trial registration:**

ClinicalTrials.gov Identifier NCT03707106.

**Supplementary Information:**

The online version contains supplementary material available at 10.1186/s13722-025-00561-2.

## Background

Despite knowledge about severe smoking-related health consequences, only 5% of spontaneous quitting attempts lead to long-term abstinence [1]. Thus, the current guidelines for smoking cessation recommend cognitive behavioral therapy (CBT) supplemented by pharmacological support [2]. However, even after therapy according to recent guidelines, the 1-year abstinence rate is unsatisfactory, at only 25–40% [3]. Therefore, our primary aim was to further improve this gold standard CBT with an add-on that specifically addresses relapse prevention. One approach that is highly discussed in the field is cue exposure therapy (CET). CET targets cue-reactivity (CR), an automated response to drug-related cues closely related to relapse [4], which is still neglected in current smoking cessation programs. Initially, neutral cues or contexts gain motivational value by means of classical and operant conditioning and elicit CR, which manifests as a physiological stress response, craving, and approach behavior [5]. During unaided smoking cessation, CR was a better predictor for relapse within a week than withdrawal symptoms or trait impulsivity [6]. CET aims at an inhibition of CR based on extinction learning [7] and habituation. Extinction learning is highly context dependent, which could explain the current moderate effects of cue exposure in the clinical setting (see, e.g., meta-analysis for alcohol use disorders [8]). To overcome the context restrictions of the clinical setting, VR scenarios can be used to generate high-risk contexts for CET [9]. Furthermore, complex social interactions can be generated in VR, allowing smokers to learn to withstand smoking offers in standardized high-risk contexts. These context effects were validated in smokers [10], indicating the ability of the VR-CET to provide a complex context for CR in smokers [11, 12]. However, in clinical application, the evidence is inconclusive. Goldenhersch et al. (2020) investigated the VR-CET using either a self-help program with VR-based mindful cue exposure and psychological advice or self-guided work with a smoking cessation manual [13]. Immediately after the intervention, abstinence rates were significantly higher in the VR-based mindful exposure group (23%) compared to the control group (5%). However, at the 90-day follow-up, abstinence rates increased to 33% in the VR-based group, while only 20% of participants in the control group provided follow-up data. Due to high dropout rates, no statistical analysis was conducted for the 90-day follow-up, limiting the interpretation of long-term effects. Pericot-Valverde et al. (2019) conducted a study closely aligned with our design, investigating VR-based cue exposure as an add-on to a cognitive behavioral therapy (CBT) smoking cessation program [14]. Various VR-based cue exposure scenarios were utilized, including offers from other smokers, alcoholic beverages, and coffee as triggers. While their intervention effectively reduced craving, no significant differences in abstinence rates were observed between groups. In fact, on a descriptive level, relapse rates were even higher in the VR group compared to the control group. To better understand these inconsistent results, interindividual differences, such as baseline cue reactivity (CR), nicotine dependence severity, and motivation to quit, should be considered as predictive variables for CET efficacy. Moderator analyses revealed that the decline in craving throughout CET was greatest in participants who displayed initial CR [15]. One potential negative predictor is the intensity of nicotine dependence according to the Fagerström Test for Nicotine Dependence (FTND), which predicts more withdrawal symptoms and poorer smoking cessation outcomes in terms of achieving initial abstinence, lapse risk, and the lapse-relapse transition [16]. Furthermore, the motivation to quit smoking predicted maintenance after smoking cessation [17].

Our primary aim was to enhance the efficacy of a well-established smoking cessation program by adding an intervention targeting CR in a virtual environment (VR-CET) and comparing it to an active control group (PMR) six months after treatment. VR offers immersive and realistic contexts targeting real-world smoking triggers, enabling participants to practice coping strategies in controlled yet lifelike scenarios. PMR training has not shown specific effects on smoking cessation in previous studies [18]. Therefore, we did not expect direct effects of PMR on craving or smoking behavior. Using PMR as an active control condition allowed us to control for unspecific effects related to the additional time spent in therapy while evaluating the specific effects of VR-CET on relapse prevention. The primary outcome was the six-month abstinence rate after a group-based state-of-the-art smoking cessation program using VR-CET for relapse prevention in comparison to unspecific relaxation training (progressive muscle relaxation, PMR [19]). The number of smoked cigarettes, 7-day point abstinence, craving, and self-efficacy were assessed as secondary measures for treatment efficacy immediately after the intervention and at the follow-up six months thereafter and were compared to baseline measures. We hypothesized that abstinence rates would be higher in the VR-CET group (primary measure) compared to the PMR group six months after the intervention. Additionally, we expected that craving and the number of smoked cigarettes would be more strongly reduced, and self-efficacy more strongly increased, in the VR-CET group compared to the PMR group at the six month follow-up. To assess the predictive value of CR, we related the maximum craving and the variance in craving during cue exposure at baseline to abstinence six months after smoking cessation. To assess whether individual add-on therapy was affected by personal attitudes toward it, smokers were asked to rate the expected efficiency of the assigned intervention.

## Methods

### Design

In this two-arm randomized controlled trial, smokers completed a baseline measurement and thereafter were assigned to either the PMR or VR-CET group [20] by the independent Biometric Institute Tuebingen which was blinded by only receiving the participants ID for further randomization without access to a reidentification list or personal contact to the participants. After baseline, group therapy started for 6 consecutive weeks, with either the PMR or VR-CET as an add-on starting in week three (see Table 1). After week six of group therapy, there was an individually scheduled post-treatment measurement. Follow-up assessments at 1 month and 3 months after group therapy were conducted via postal questionnaires. The 6-month follow-up assessment took place on-site at the University Hospital Tuebingen or University of Regensburg, specifically in the laboratory where baseline and post-treatment CR measurements were conducted.

**Table 1:** Smoking-related sample characteristics at baseline (ITT analysis)

	VR (*n* = 122)	PMR (*n* = 124)	Statistics
Sex	♂ *n* = 69 (57%) ♀ *n* = 53 (43%)	♂ *n* = 68 (55%) ♀ *n* = 56 (45%)	*Chi*^*2*^ = 0.074, *p* =.786, OR(PMR/VR) = 0.93
Age	*M* = 46.3 (*SD* = 13.4)	*M* = 47.8 (*SD* = 13.4)	*T*_*244*_ *= 0.919*, *p =.359*
Employment	Student: *n* = 12 Worker *n*= 9 Employee: *n*= 66 Civil servant *n* = 3 Self-employed *n*= 8 Retired: *n* = 12 Unemployed: *n* = 5 Missing: *n* = 7	Student: *n* = 12 Worker *n* = 5 Employee: *n* = 62 Civil servant *n* = 10 Self-employed *n* = 14 Retired: *n* = 14 Unemployed: *n* = 2 Missing: *n* = 5	*Chi*^*2*^ *= 6.62*, *p =.251*
BDI	*M* = 3.1 (*SD* = 2.9)	*M* = 4.0 (*SD* = 3.9)	*Z* = 1.42, *p* =.157
FTND	*M* = 4.7 (*SD* = 2.1)	*M* = 4.8 (*SD* = 2.1)	*Z* = 0.054, *p* =.957
Cigarettes per day	*M* = 18.6 (*SD* = 6.2)	*M* = 19.0 (*SD* = 7.6)	*Z* = 0.098, *p* =.922
Medical advice to quit	66 (48%)	72 (58%)	*Chi*^*2*^ = 0.308, *p* =.579, OR(PMR/VR) = 0.87
Previous quitting attempts	101 (84%)	104 (84%)	*Chi*^*2*^ = 0.007, *p* =.933, OR(PMR/VR) = 0.97

The study was approved by the Ethics Committee of the Faculty of Medicine at the University Hospital of Tuebingen (no. 836/2016BO1) and by the Ethics Committee of the German Psychological Society (DGPs) for the University of Regensburg (no. AM022017), and written informed consent was given by all participants in accordance with the Declaration of Helsinki in its latest version. The study was preregistered (ClinicalTrials.gov Identifier: NCT03707106) on 16.08.2018, before recruitment started.

### Participants

**Fig. 1:**
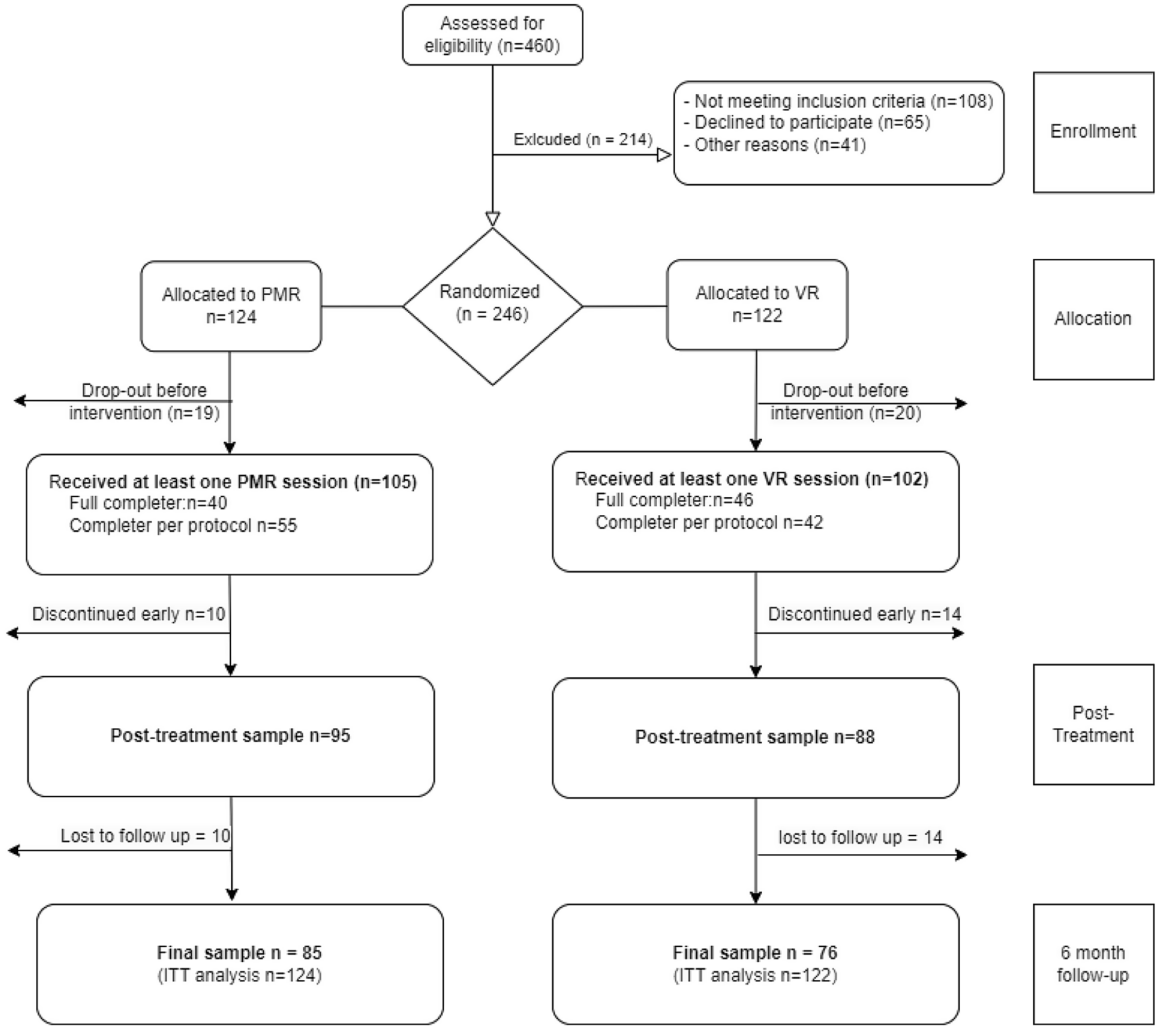
Flow-Chart

Overall, *N* = 246 daily smokers were eligible for randomization, meeting the inclusion criteria (age 18 (legal smoking age), daily smoking of at least 10 cigarettes and a minimum of 2 years of smoking) and exclusion criteria (no pregnancy, no participation in a smoking cessation program within the last 6 months, no current acute psychiatric diagnosis other than tobacco dependence (F17.2), and no lifetime diagnosis of psychosis, PTSD, or bipolar disorder). The sample size calculation was based on prior studies [21], which reported 70% abstinence immediately after CBT and 30% at the 6-month follow-up in the control group (CBT + PMR). Since no efficacy data for VR-CET were available during planning, abstinence rates were estimated at 80% post-intervention and 50% at follow-up. With alpha = 0.05 and beta = 0.80, the PS software determined a required *n* = 93 per group. Adjusting for a 20% drop-out rate, the final sample size was set at *n* = 120 per group. Smokers were recruited via mailing lists at the Universities and University Hospitals of Tuebingen and Regensburg, as well as through announcements in the local press. Participants were informed about the study procedures in a group setting, with any remaining questions clarified during an individual interview where the inclusion criteria were assessed. Table 2 presents the sample characteristics, while Fig. 1 reveals the flow-chart for the study.

**Table 2:** Structure and content of the Group-Based Cognitive-Behavioral smoking cessation (CBT) program (“Nichtraucher in 6 wochen” [Nonsmoker in six weeks]). For a detailed description of the four different VR scenarios, see [47])

Measure	Intervention	VR	PMR
Baseline	None	Cue reactivity assessment (5 min control condition and 5 min smoking cue exposure)	
Week 1 CO	Strengthening abstinence motivation, observing current smoking patterns (115 min group-based)		
Week 2 CO	Preparation of quit smoking day, developing coping strategies for situations associated with smoking, explanation of add-on therapy (120 min group-based)		
**Quit day**			
Week 3 CO	Development of alternative behaviors to smoking (90 min group-based)		
	Add-on	*- Rumination/loneliness scenario (18 min)* *- Party scenario 13 min*	*Long PMR version + short version (total 40 min)*
Week 4 CO	Focus on positive aspects of abstinence, analysis of high risk situations for relapse		
	Add-on	- Stress scenario (17 min) - Café scenario (16 min)	*Long PMR version + short version (total 40 min)*
Week 5 CO	Therapy agreement for relapse prevention		
	Add-on	*- Rumination/loneliness scenario (18 min)* *- Party scenario 13 min*	*Long PMR version + short version (total 40 min)*
Week 6 CO	Relapse crisis plan		
	Add-on	- Stress scenario (17 min) - Café scenario (16 min)	*Long PMR version + short version (total 40 min)*
Post-Treatment CO	None	*Cue reactivity assessment (5 min control condition and 5 min smoking cue exposure)*	
6-Month Follow-up CO	None	*Cue reactivity assessment (5 min control condition and 5 min smoking cue exposure)*	

### Procedure

Participants were invited to participate in a baseline measurement, including questionnaires and an EEG measurement, to capture the biological correlates of smoking CR (Kroczek et al., *in preparation*). The predictive value of EEG CR measures in the VR group will be discussed elsewhere. After this baseline measurement, each participant was randomly assigned to either the smoking cue exposure (VR-CET) group or the progressive muscle relaxation (PMR) group by the Biometrics Institute Tübingen. Appointments for a new group of the smoking cessation program were scheduled as soon as at least six smokers had confirmed participation. All participants received a well-established group-based cognitive-behavioral SC program (“Nichtraucher in 6 Wochen [Nonsmoker in six weeks]” [21, 22]) consisting of six weekly evening sessions in fixed groups of 6–8 participants, with either VR or PMR as add-on intervention starting from week 3 (see Table 2 for content). After six weekly group-based SC sessions, individual appointments were scheduled for EEG post-treatment measurements. Follow-up at 1 month and 3 months after group therapy was assessed postally via questionnaires, and the follow-up at 6 months after group therapy was conducted at the University Hospital Tuebingen or the University of Regensburg, in the laboratory where Baseline and Post-treatment measurements of cue-reactivity took place. No Virtual Reality (VR) sessions were conducted during follow-ups; measurements at all time points were identical for both groups.

### Add-on therapies

#### Smoking cue exposure therapy in virtual reality (VR-CET)

Four different scenarios were implemented [20, 23] targeting (1) loneliness and ruminating about problems (duration 18 min), (2) a social party situation (13 min), (3) a social situation in a café (16 min), and (4) a social stress situation, evoked by the Trier Social Stress Test (17 min), with each scenario being repeated twice. At each appointment that included a VR session, two scenarios were combined. In this way, with consideration of the mounting and adjustment of the VR glasses, each VR session lasted approximately 40 min. Two VR environments were created with Unreal Engine 4 (Epic Games, Raleigh, USA) by VTplus GmbH (Würzburg, Germany), and two were created at the Department of Psychology (Clinical Psychology and Psychotherapy) at the University of Regensburg using the Steam Source engine (Valve Corporation, Bellevue, Washington, USA). All scenarios were presented using a VIVE Pro head-mounted display (HMD; HTC Corporation, Taoyuan, Taiwan). To individualize the stimuli in the scenarios, participants could select their preferred tobacco brand from a list of 21 brands, and the chosen brand design was used for all VR scenarios.

Two VR systems were available at each study center; therefore, the participants had to wait for the VR-based cue exposure (two concurrent sessions were possible either before or after the group sessions). The waiting time for participants was rotated between sessions. The participants completed the VR sessions individually, with an experimenter present who controlled the VR with the software Cybersession Research (VTplus GmbH, Würzburg, Germany). The participants’ views were duplicated for the investigator on a separate computer monitor.

### Progressive muscle relaxation

PMR was conducted in a seated upright position in a group-based setting and was carried out at the end of the sessions to avoid waiting time. Participants were instructed to perceive tension and relaxation in all body areas beginning with the hands, followed by the arms, facial muscles, neck, shoulders, torso, and legs [19]. Thereafter, participants repeated a second short PMR version. The total duration of PMR was approximately 40 min per session in order to match the duration of the VR-CET.

## Measures

### Primary outcome measure

The primary outcome measure was continuous abstinence according to the Russell Standard [24], with fewer than five smoked cigarettes altogether according to self-reports and biochemical validation by a CO measurement (cutoff 10 ppm, piCO Smokerlyzer, Bedfont, England). The primary outcome was assessed 6 months after group therapy in the same laboratory rooms as baseline and post-treatment measurements took place. CO measurements could be conducted even through the pandemic, after implementing enhanced hygienic measures, like ensuring proper sanitization techniques and the study nurse wearing a face mask.

### Secondary outcome measures

As secondary measures, we assessed the 7-day (point) prevalence of abstinence and the number of smoked cigarettes in the last week of group therapy as well as one, three, and six months thereafter. Smoking-related self-efficacy was measured by the German Smoking Abstinence Self-Efficacy Scale (SES, Cronbach’s Alpha = 0.95 [25]). Craving was assessed by the German version of the brief Questionnaire of Smoking Urges (QSU-b, Cronbach’s alpha = 0.97 [26]. Both SES and QSU-b were assessed prior to treatment, in the first and last weeks of the SC program, and one, three, and six months thereafter.

### Baseline predictors of abstinence

FTND [27] was used to quantify the severity of nicotine dependence, distinguishing light (FTND 0–2), moderate (FTND 3–5), and heavy smokers (FTND 6–10). To evoke CR, a previously validated smoking cue-exposure task composed of a gradually increasing intensity of confrontation with each individual’s cigarettes and smoking paraphernalia was used [28]. From 5 verbal craving ratings during baseline cue exposure, individual craving changes (differences between consecutive craving ratings summed) and maximum cravings (highest value of the 10 baseline ratings) were extracted as predictors [29]. Motivation for smoking cessation was assessed by summing the three ratings of importance, confidence, and decision. Belief in personal benefit on the VR-CET/PMR was rated on one item (not at all (1) - very much (10)). The exact German wording can be found in Supplement 2.

### Statistical analyses

#### Primary outcome measures

Statistical analyses were conducted with IBM SPSS Statistics 21. A chi-squared test was used for the primary outcome to compare the abstinence rates between the VR-CET and PMR groups. According to an intention-to-treat (ITT) analysis, drop-outs were considered smokers in this analysis.

### Secondary outcome measures

A chi-squared test was used to assess differences between groups in the 7-day point abstinence one month, three months and 6 months after treatment, respectively. Independent t-testing was applied to test for significant group differences in the number of smoked cigarettes (only for individuals that were relapsed). SES and QSU-b scores were analyzed with a 6 (time) × 2 (add-on therapy: PMR, VR) repeated-measures ANOVA. In a subgroup analysis of therapy-adherent participants, only participants who had accomplished smoking cessation when starting add-on therapy (CE/PMR) were included in the repeated-measures ANOVA analyzing group-dependent changes in self-efficacy and craving ratings. This approach was used as abstinence is prerequisite for relapse prevention. Missing values in the QSU and SES needed to be imputed with only 131 complete datasets (using the R “MICE” package, *multiple imputation for chained equations*). FTND was included as a between-subject factor reflecting smoking severity.

### Binomial regression

The predictive value of severity of nicotine dependency (FTND), baseline CR (craving change and maximum craving), smoking cessation motivation, and expected efficacy of the add-on intervention at baseline on the primary measure (continuous abstinence) was analyzed via binomial logistic regression.

## Results

### Sample and drop-outs

A total of 246 smokers were randomized to either VR-CET (*n* = 122) or PMR add-on therapy (*n* = 124). At the 6 month follow-up, data was available for *n* = 85 (PMR) and *n* = 76 (VR), see Fig. 1 for a flow chart. Within the ITT approach for the primary outcome, individuals without available data were counted as relapsed.

For an additional post hoc analysis, only smokers who quit smoking and had not relapsed before the day of add-on therapy after smoking cessation (between weeks 2 and 3) were included, as validated by CO measurements < 10 ppm (therapy-adherent subsample), reducing the sample to 80 (*n* = 42 VR, *n* = 38 PMR).

### Primary outcome measure: abstinence

A total of 24% (*n* = 58) of the randomized smokers were abstinent 6 months after the completion of SC, with no significant effect of the add-on group for the ITT (PMR: 34/124 (24%), VR: 24/122 (20%), 𝜒^2^ = 2.05, *p* =.152, *d* = 0.18) or the analysis of available data (see Fig. 1). For the therapy-adherent subsample (abstinence during the relapse prevention), there was no significant difference in the primary outcome measure, 𝜒^2^ = 1.75, *p* =.186, *d* = 0.32.

### Secondary outcome measures: 7-day abstinence and number of cigarettes

There was no effect of add-on therapy on 7-day point abstinence or the number of smoked cigarettes for any of the three follow-up measurements (see Table 3). The mean number of smoked cigarettes (overall) decreased one month after the intervention (*M* = 9.56, *SD* = 6.55) compared to baseline (*M* = 18.90, *SD* = 6.11), t_71_ = 12.69, *p* <.001, *d* = 1.48. From one to three months (*M* = 10.79, *SD* = 6.07), the mean daily number of cigarettes increased, *t*_55_ = 3.10, *p* =.002, *d* = 1.33, and further increased to six months after therapy (*M* = 12.53, *SD* = 6.63), *t*_55_ = 3.09, *p* =.001, *d* = 0.99.

**Table 3:** Secondary outcome measures

**1 month after smoking cessation (assessment via mail)**			
	**VR** (*n* = 68)	**PMR** (*n* = 80)	*Statistics*
7-day abstinence	40 (59%)	49 (61%)	*Chi*^*2*^ = 0.09, *p* =.866,
Smoked cigarettes for *n* = 56 smokers	*M* = 10.1(*SD* = 6.5)	*M* = 9.1 (*SD* = 6.8)	*T*_*54*_ = 0.56, *p* =.577
**3 months after smoking cessation** (assessment via mail)			
	**VR** (*n* = 64)	**PMR** (*n* = 78)	*Statistics*
7-day abstinence	26 (41%)	44 (56%)	*Chi*^*2*^ = 3.50, *p* =.061
Smoked cigarettes	*M* = 10.5(*SD* = 6.3)	*M* = 10.7 (*SD* = 6.4)	*T*_*63*_ = 0.19, *p* =.849
**6 months after smoking cessation**			
	**VR** (*n* = 76)	**PMR** (*n* = 85)	*Statistics*
7-day abstinence	27 (36%)	36 (42%)	*Chi*^*2*^ = 0.785, *p* =.421,
Smoked cigarettes	*M* = 3.1(*SD* = 2.5)	*M* = 2.8 (*SD* = 0.6)	*T*_*93*_ = 0.85, *p* =.400

### Secondary outcome measure: QSU (smoking urges)

**Fig. 2:**
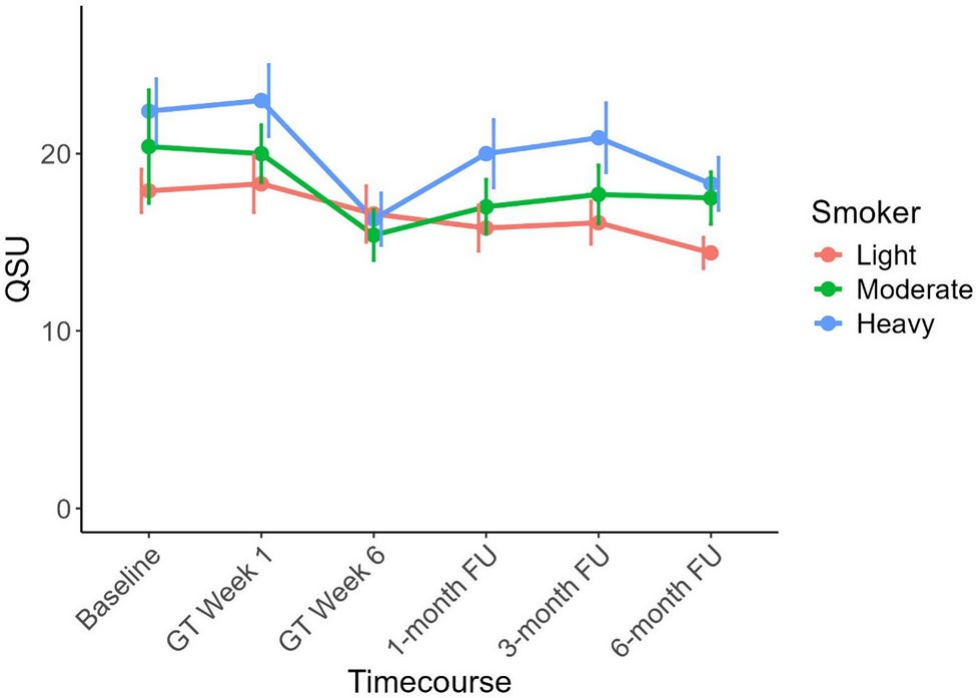
Changes in craving ratings (Questionnaire of Smoking Urges, QSU) from baseline (BL) to treatment weeks 1 and 6 as well as the three follow-up (FU) assessments (main effect based on the ANOVA results). Three separate lines depict smoker groups (according to FTND), with vertical lines indicating the standard deviation (SD). The x-axis shows the sum of the QSU scores

For craving (QSU), there was a significant main effect of “time” (*F*_3, 251_=26.309, *p* <.001, η^2^_*p*_ = 0.37; see Fig. 2) but no group interaction. Even in the therapy-adherent subsample, time had the only significant effect (*F*_4, 988_=9.079 *p* <.001, η^2^_*p*_ = 0.23).

While there was no difference in the QSU between baseline (*M* = 21.07, *SD* = 10.65) and treatment week 1 (M = 21.24, SD = 11.83, *t*_*245*_ = 0.21, *p* =.418), the QSU decreased significantly between week 1 and treatment week 6 (M = 16.10, SD = 9.20, *t*_*245*_ = 5.81, *p* <.001). The QSU values then increased from week 6 to the one-month follow-up assessment (M = 18.39, SD = 11.00, *t*_*245*_ = 2.88, *p* =.02), remained stable at the 3-month follow-up assessment (M = 19.17, SD = 11.28, *t*_*245*_ = 1.15, *p* =.125), and then decreased again between the 3-month and 6-month follow-ups (M = 18.35, SD = 8.84, *t*_*245*_ = 2.72, *p* =.003). There was no main effect or interaction with add-on therapy (*F*_4, 988_=1.4, *p* =.220, *η*^2^ = 0.006), but there was a main effect of “smoking severity” (*F*_4, 988_=4.081 *p* =.018, *η*^2^ = 0.033).

### Secondary outcome measure: SES (smoking-related self-efficacy scale)

**Fig. 3:**
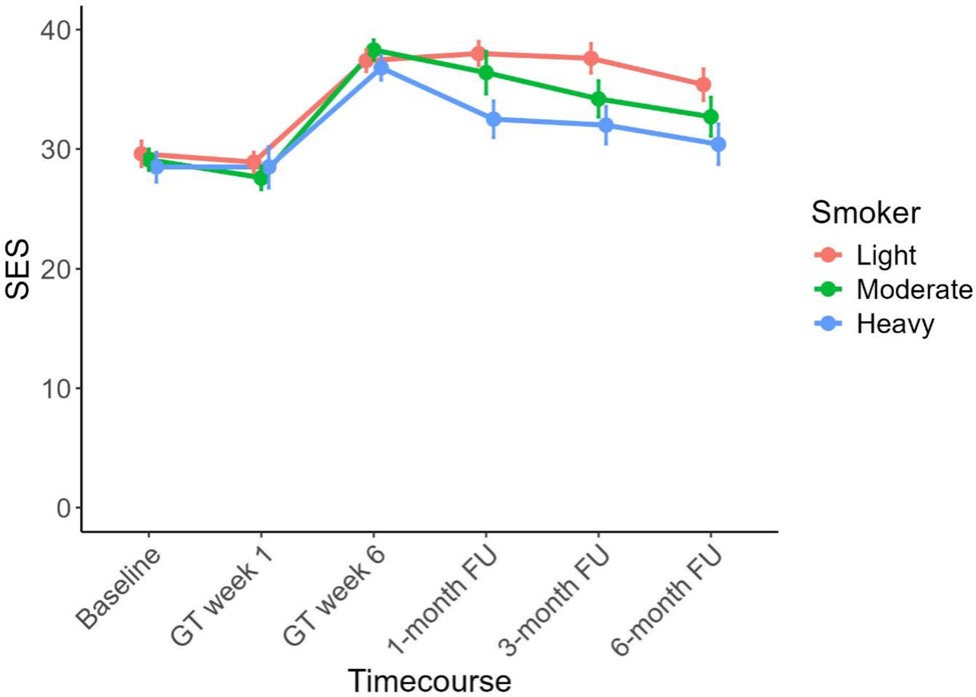
Changes in smoking-related self-efficacy scale (SES) ratings from baseline to treatment weeks 1 and 6 as well as the three follow-up (FU) assessments (significant interaction based on the ANOVA results). The vertical lines indicate the standard deviation (SD) for each measurement

For smoking self-efficacy (SES), significant main effects of time (*F*_4, 850_=49.40 *p* <.001, *η*^2^ = 0.17) and smoking severity (*F*_1, 239_=3.69 *p* =.026, *η*^2^ = 0.30) were observed. Furthermore, there was an interaction between both factors (*F*_7, 850_=2.73 *p* =.008, *η*^2^ = 0.022; see Fig. 3). Following up this interaction, the SES of light smokers was significantly greater than that of heavy smokers at the one-month (*t*_116_ = 4.47, *p* <.001), 3-month (*t*_95_ = 3.96, *p* <.001), and 6-month follow-up measurements (*t*_96_ = 3.26, *p* =.002). SES was increased in light smokers compared to moderate smokers only at the 3-month FU (*t*_105_ = 2.07, *p* =.021) (6 months: *t*_105_ = 1.52, *p* =.066). No significant differences were found between moderate smokers and heavy smokers.

### Predictors of the primary outcome measure

Within the VR group, a model with maximum craving and craving changes during baseline cue exposure, FTND, motivation, and the expectancy of the personal benefit of the VR-CET predicted abstinence as the primary outcome significantly only for maximum craving and the expectancy of personal benefit (𝜒^2^ = 11.13, *p* =.025, *R*^2^=0.157 [weak effect]). Maximum craving was negatively associated with abstinence, and expectancy was positively associated with abstinence. The model classified 77% correctly; the parameters can be found in Table 4. The same model was not significant in the PMR group (𝜒^2^ = 3.28, *p* =.657, *R*^2^ = 0.043). For the therapy-adherent subsample, the model was again significant in the VR group (𝜒^2^ = 14.57, *p* =.025, *R*^2^ = 0.360). However, only craving change (ß = 0.032) and maximum craving (ß =-0.059) were significant predictors.

**Table 4:** Regression parameters for the primary outcome measure (6-month abstinence) in the VR group (significant predictors are highlighted in bold)

	*B*	*SE*	*Wald*	*p*	*Exp(B)*	*CI for EXP(B)*
FTND	− 0.140	0.125	1.24	0.265	0.870	0.680–1.122
Craving change	0.010	0.007	2.15	0.143	1.010	0.997–1.003
**Maximum craving**	**− 0.031**	**0.014**	**5.20**	**0.023**	**0.969**	**0.943–0.996**
**Belief in VR-CET**	**0.468**	**0.179**	**6.88**	**0.009**	**1.597**	**1.126–2.266**
Motivation	− 0.153	0.085	3.22	0.073	0.858	0.726–1.014

## Discussion

Contrary to our hypothesis, the additional cue exposure component in virtual reality did not increase abstinence rates compared to nonspecific relaxation training supplemented with a validated smoking cessation program. This needs to be discussed in light of evidence of the prognostic value of craving at baseline for abstinence as the primary outcome in the VR-CET group: lower maximum craving predicted a positive therapeutic outcome. One implication of this result is that smokers experiencing high levels of cue reactivity (CR) at baseline may not have received enough cue exposure sessions. Other authors with four sessions [30, 31] did not find significant craving reductions in smokers either. While positive effects of VR-CET could be found, the corresponding studies included ten [32] sessions in smokers or eight VR-CET sessions in patients with alcohol dependency [33]. Our own data indicate that four sessions of CET can be enough for smokers with low levels of baseline craving but should be increased for smokers with high baseline craving. It is important to consider that the severity of nicotine dependence (FTND) was also involved in the regression but did not predict abstinence, indicating that baseline craving is an independent measure. There is also previous evidence that craving during withdrawal and smoking severity predict relapse [34]. In our data, baseline craving ratings were independent of withdrawal, as smokers in our RCT smoked as usual while smoking cessation was being prepared in group-based cognitive behavioral therapy in sessions one and two, with smoking cessation being initiated between weeks two and three. In a post hoc analysis, a therapy-adherent subsample was defined by CO values below 10 in the measurements during group therapy sessions 3–6. According to our design, every smoker assigned to the VR group took part in the CET for relapse prevention, irrespective of the therapy adherence defined by smoking cessation. However, the predictive value of maximum craving even increased in this subsample. However, this points to the importance of individual interventions that should be considered even in RCTs. A possible approach is to work individually with smokers who have not yet reached abstinence before proceeding with the assigned relapse prevention interventions, along with the cost of reduced standardization.

The German treatment guidelines recommend that “behavioral treatments to support tobacco abstinence should include several components (especially psychoeducation, motivation reinforcement, measures for short-term relapse prevention, interventions to strengthen self-efficacy, everyday practical counseling with concrete behavioral instructions, and practical coping strategies (problem-solving and skills training, stress management))” [2]. Cue exposure is not recommended in these guidelines and is not carried out systematically. The current trial, therefore, aimed to investigate the efficacy of a low-dose cue-exposure treatment as an adjunct to guideline-based tobacco cessation treatment and revealed the necessity of assessing the prerequisites for the intervention and allowing an individualized decision for its adaptation. This study examined the efficacy of VR-CET in comparison to PMR, both as a supplement to a cognitive behavioral smoking cessation program that has already been published and proven to be effective [21, 22], with high standards related to RCTs.

Although it would have been a strength if hypotheses had been confirmed, the strong control group is a limitation for the interpretation of the results, as it could be a possible contributor to the unexpected effects. In the case of PMR, we used a control intervention without known specific effectiveness in smoking cessation, yet a subgroup of smokers may have benefited from this intervention as an aid in coping with relapse-prone situations. Involving more specific characteristics than the FTND, e.g., impulsivity, depression, and sensation seeking [35], could provide more features for the decision to apply the respective additional therapy. Mechanisms underlying abstinence in both conditions may differ, despite yielding comparable overall abstinence rates. Preliminary evidence suggests that PMR may reduce cravings, supporting its potential role as an adjunct treatment for relapse prevention in smokers [36]. This implies that the control group in our study may have benefited from stress reduction as a nonspecific factor, which could have contributed to relapse prevention and diminished the differences to VR-CET targeting craving reduction.

In contrast, a study by Malbos et al. (2023) comparing VR smoking cue exposure with cognitive behavioral therapy found significant differences in abstinence rates [37]. However, their results reported only post-intervention abstinence rates, with 68% abstinence observed in the VR-CET group. While these findings are promising, further research with longer follow-up periods (at least 6 months) is required to validate their reliability. Consistent with our earlier discussion, Malbos et al. allowed participants to individually select VR-CET scenarios, and their protocol included six VR sessions within an eight-session intervention—considerably more extensive than the four sessions implemented in our study. This difference in session frequency may partly account for variations in effectiveness and underscores the need for further research to identify optimal intervention designs.

A benefit of our RCT was the representative sample of smokers with characteristics typical of such a study design: more male smokers, a mean FTND score of approximately 4.5 points, an average daily consumption of 20 cigarettes, and a high proportion of participants (84%) with at least one previous quit attempt. The primary outcome was abstinence according to the “Russell Standard” definition [24]. The average achieved abstinence rates were good compared to the average long-term success rate stated in the S3 treatment guidelines, taking into account the absence of concomitant medication. Other recent studies on smoking cessation, such as studies on hypnotherapy [38] or mindfulness-based procedures [39], have reported similar abstinence rates of up to 20–25%. In line with such results, our earlier study [40] revealed similar abstinence 6-months after CBT (1-year abstinence: 21.2%) and hypnotherapy (16.7%). Unfortunately, no further treatment components could improve the established interventions so far [41]. Based on our data, a possible explanation for the insufficient efficacy of cognitive behavioral interventions in individuals with addiction could be the lack of individualization of the interventions. This affects not only the number of sessions but also the time course of application.

A future approach for the treatment of addiction is to consider motivational consumption states, tailoring the assignment to an investigated intervention more individually. This approach is promising for demonstrating an additional effect of the applied treatment component that our study could not show for our primary outcome. Likewise, for the secondary measures, there was no specific effect of the additional treatment. Nevertheless, the overall number of smoked cigarettes and the desire to smoke decreased, while self-efficacy increased. Fluctuations throughout the observed period still show improvements at the end compared to baseline measures, an effect that can essentially be explained by the successfully treated individuals. Another explanation for the lack of effect of VR-CET compared to nonspecific treatment is that the heavy smokers did not receive any medication, which is suggested in the guidelines indicating/recommending combination therapy. A large-scale study in 1843 smokers receiving cognitive behavioral therapy for smoking cessation revealed a 21% abstinence rate at a 12 month follow-up, which is comparable to our abstinence rates. Abstinence rate was 33% in the group that received additional nicotine replacement therapy [42]. Thus, without medication, heavy smokers in particular are likely to experience more withdrawal symptoms increasing relapse risk. Since the VR training started one week after smoking cessation, these withdrawal symptoms might have affected the effectiveness of the cue exposure treatment. Again, an individual approach for an RCT could overcome such limitations.

Based on principal learning theories, smokers who experienced intense craving were expected to profit from VR-CET, which was not confirmed by our data. We already discussed the low number of repetitions of VR-CET sessions and the lack of withdrawal medication to be related to this lack of expected effects. Compared to other fields where exposure treatment is applied, e.g., patients with posttraumatic stress disorder (PTSD), there are additional instructions to apply emotion regulation skills during exposure [43]. Therefore, a possible shortcoming of our VR-CET could be the lack of available skills to handle the CR-evoking scenario. In future studies, inhibitory control during CET could be supported by noninvasive brain stimulation, an approach that has already been tested during exposure in patients with PTSD [44]. Furthermore, another enhancement of the VR-CET could be the application of emotion regulation strategies for craving during sessions. Another approach is the use of biofeedback to regulate the intensity of VR-CET, as the physiological response is another important level of CR that is not completely captured by craving [45].

Last but not least, the study was influenced by the COVID-19 pandemic (2020/2021): Compared to previous smoking cessation studies by our working group [21, 35], the follow-up response rates were significantly lower. In the course of the conservative ITT, these participants were considered relapsing smokers. From March 2020 onward, both the smoking behavior of the participants and the technical processes of the study, such as recruitment and contacting the participants, were affected by the pandemic situation. International data on smoking during the pandemic were mixed [46], while German data (DEBRA study) show a surprising increase in smoking behavior and a decrease in smoking cessation success during the pandemic, despite the known effects of smoking on lung damage (https://www.debra-study.info/). There is evidence that relapse rates are particularly high among smokers with high stress levels [38]. Therefore, increased COVID related stress levels might have affected abstinence rates in our sample.

Furthermore, the rapid development of VR technology must be considered. Realistic scenarios are the goal for increasing the efficacy of VR-CETs; therefore, it needs to be considered whether technical improvement in quality could increase their efficacy. Subjective ratings of the four scenarios are reported elsewhere [23], and increases in subjective presence could be another future direction for improving CET efficacy.

Despite these limitations, the high methodological standards of our trial were comparable to those of pharmacological studies in terms of external monitoring and data management, a safety assessment, predefined analysis plans and blinded follow-ups. Four VR scenarios were established and tested in this large study sample, where acceptance of the VR-CET among smokers was shown.

In conclusion, we were able to demonstrate the relevance of CR to smoking cessation, but the specific parameters of our CET – such as duration, character and intensity – may not have been adequate to sufficiently change CR, e.g., an insufficient number of training repetitions. Future directions in VR incorporate further sensory modalities such as olfactory or haptic information that need to be investigated regarding a potential increase in the efficacy of cue exposure. In our scenarios, smelling or even drinking coffee or alcohol could intensify the scenarios. Furthermore, the presentation of additional high-risk situations (e.g., an emotional conflict to experimentally induce rumination) in VR could be used to broaden the scope of processes and situations strongly related to relapse that are addressed in VR-CETs.

There are other important parameters that need to be considered in future studies, including the number and frequency of cue exposure sessions and longer or more adaptive protocols that include personal high-risk smoking situations, internal or emotional cues or craving-adapted session protocols. Furthermore, an individualized approach based on baseline CR, self-efficacy, and coping strategies might significantly improve VR-CET.

## Electronic supplementary material

Below is the link to the electronic supplementary material.

Supplementary Material 1

## Data Availability

The data that support the findings of this study are not openly available due to reasons of sensitivity and are available from the corresponding author upon reasonable request. Data are located in controlled access data storage at University Hospital Tuebingen.

## Abbreviations

BL: Baseline
CBT: Cognitive behavioral therapy
CET: Cue Exposure Therapy
CO: Carbon monoxide
CR: Cue Reactivity
EEG: Electroencephalography
FTND: Fagerström test for nicotine dependence
FU: Follow-up
G-CBT: Group Cognitive Behavioral Therapy
ITT: Intention-to-Treat
PMR: Progressive Muscle Relaxation
QSU: Questionnaire of Smoking Urges
RCT: Randomized controlled trial
SES: Smoking-related Self-efficacy Scale
VR: Virtual Reality
