# Supplementary material for: Virtual reality cue exposure as an add-on to smoking cessation group therapy: a randomized controlled trial
Source: Addict Sci Clin Pract. 2025 Apr 11;20:34. doi: 10.1186/s13722-025-00561-2 (PMC11987342; doi:10.1186/s13722-025-00561-2)
Supplement: Supplementary file 1 — Supplementary Material 1 [file 13722_2025_561_MOESM1_ESM.docx]

**Supplementary Material**

Supplement 1: Procedure and results of the prestudy to identify suitable high-risk situations for relapse.

To identify suitable high-risk situations for relapse, we conducted a literature search (Buczkowski et al., 2014; Cummings et al., 1985; Pisinger et al., 2011), resulting in a questionnaire describing nine high-risk situations that could be implemented in VR. Additional open questions allowed smokers to describe individual high-risk situations or to propose changes to situations that have already been described that could increase the likelihood of relapse. The questionnaires were completed by an independent group of smokers (n=17) participating in a smoking cessation program that is regularly offered at the University Hospital Tuebingen. Each scenario was rated considering its risk for relapse (0–100%). We calculated mean values for every scenario across participants, which indicated the four highest ranked scenarios we subsequently used to script the VR-based cue exposure. For this purpose, screenplays were written, and videos of the scenarios in which students played the roles of the VR agents were created as a guideline for the preparation of the VR environments.

In addition to the four scenarios we finally selected for the main study, five other scenarios were described in the questionnaire: angry, waiting, beer garden, success, and boredom. However, participants rated these scenarios as less relapse-related. In Table S1, the mean relapse risk ratings are listed for all nine scenarios. The scenarios that were ultimately not selected were described in the questionnaire as follows (see the main article for the four selected scenarios):

*Being angry*: “Try to think as intensely as possible about a current issue from your everyday life that annoys you and makes you angry. Then, imagine: You are standing in the smoking area of a pub, in front of you on the table are alcoholic drinks, including yours. Additionally, an ashtray, as well as another guest's cigarettes and a lighter are on the table. The other guest offers you a cigarette several times.”

*Waiting*: “Imagine you have an important appointment and are dependent on public transportation to get there. Your bus finally arrives at the station with some delay, and you rush to the platform in a great hurry. Then, imagine: Once there, all you see is your train just leaving the station. The next suitable connection does not leave until a whole hour later, so it is almost impossible for you to get to your appointment on time. Three other passengers who were on the bus with you angrily move to the smoking area right next to you and light a cigarette. One of them offers you a cigarette several times.”

*Beer garden*: “You are sitting together with five other people in a beer garden. There is drinking and laughing, and the mood is cheerful. After everyone has finished eating and a waiter has cleared away the dishes, three of the other people light a cigarette. One of the smokers offers you a cigarette several times.”

*Success*: “Try to imagine a sense of success from your everyday life as intensely and pictorially as possible. Then, imagine: You have just completed an interview (e.g., as part of a hiring process) and receive feedback that you more than meet expectations and you get the job. On this celebratory occasion, an interviewer suggested that you smoke a cigarette together and offer you one several times.”

*Boredom*: “Imagine you have had a hard day and all you want to do is get home. However, you are dependent on the bus. Then, imagine: When you arrive at the bus stop, you realize that the next bus will not leave for another 20 minutes. Other waiting passengers are smoking with relish and talking about the new packaging design of their favorite brand. They say that the taste, on the other hand, has remained the same; you can rely on it. You think about asking the smokers for a cigarette to bridge the boring waiting time a little.”

Table S1 – Rating of relapse risk (range 0–100; means and standard deviations) of nine possible VR scenarios (questionnaire-based prestudy; N=17)

| **Szenario** | ***M* [%]** | ***SD*** |
| --- | --- | --- |
| Loneliness and rumination | 50 | 38 |
| Party | 48 | 39 |
| Café | 37 | 30 |
| Stress | 33 | 38 |
| Being angry | 31 | 37 |
| Waiting | 28 | 33 |
| Beer garden | 26 | 29 |
| Success | 19 | 30 |
| Boredom | 19 | 29 |

Supplement 2:

Assessment of **belief in VR-CET (belief in PMR** for the respective model**):** (rating from 1-10)

“Wie überzeugt sind Sie davon, dass **Sie persönlich** es mit Hilfe des Gruppenprogramms inklusive des **virtuellen Abstinenz-Trainings** schaffen würden, das Rauchen erfolgreich aufzugeben"

- “How confident are you, that you will personally succeed in smoking cessation by means of the group program including the virtual abstinence training?”

“Wie überzeugt sind Sie davon, dass **Sie persönlich** es mit Hilfe des Gruppenprogramms inklusive der **Progressiven Muskelrelaxation (nach Jacobsen)** schaffen würden, das Rauchen erfolgreich aufzugeben"

- “How confident are you, that you will personally succeed in smoking cessation by means of the group program including progressive muscle relaxation?

Assessment of **SC motivation:** (rating from 1-10) → sum of these 3 items

“Wie wichtig ist es Ihnen im Moment, rauchfrei zu werden/bleiben?"

- “How important is it to you at the moment to become/stay smoke-free?”

“Wie zuversichtlich sind Sie, dass Sie es schaffen können, rauchfrei zu werden/bleiben”

- “How confident are you at the moment to become/stay smoke-free?”

“Wie entschlossen sind Sie im Moment, rauchfrei zu werden/bleiben”

- “How determined are you at the moment to become/stay smoke-free?”
